# Zhurkov’s Stress-Driven Fracture as a Driving Force of the Microcrystalline Cellulose Formation

**DOI:** 10.3390/polym12122952

**Published:** 2020-12-10

**Authors:** Sergey V. Stovbun, Mariya G. Mikhaleva, Aleksey A. Skoblin, Sergey V. Usachev, Sergey N. Nikolsky, Vasily A. Kharitonov, Kseniya I. Kovaleva, Galina G. Politenkova, Alexander S. Vedenkin, Dmitry V. Zlenko

**Affiliations:** 1 N.N. Semenov Institute of Chemical Physics, RAS, Kosygina 4, 119991 Moscow, Russia; s.stovbun@yandex.ru (S.V.S.); wawe@bk.ru (M.G.M.); ab1954@yandex.ru (A.A.S.); usachevsv@inbox.ru (S.V.U.); nikolskij56@mail.ru (S.N.N.); vch.ost@mail.ru (V.A.K.); kovaleva_kseniya@bk.ru (K.I.K.); politenkova_g@mail.ru (G.G.P.); a.s.vedenkin@gmail.com (A.S.V.); 2 Faculty of Biology, M.V. Lomonosov Moscow State University, Lenin Hills 1/12, 119192 Moscow, Russia

**Keywords:** microcrystalline cellulose, nanocellulose, acid hydrolysis, mechanical stress

## Abstract

Microcrystalline cellulose (MCC) is a chemically pure product of cellulose mechano-chemical conversion. It is a white powder composed of the short fragments of the plant cells widely used in the modern food industry and pharmaceutics. The acid hydrolysis of the bleached lignin-free cellulose raw is the main and necessary stage of MCC production. For this reason, the acid hydrolysis is generally accepted to be the driving force of the fragmentation of the initial cellulose fibers into MCC particles. However, the low sensibility of the MCC properties to repeating the hydrolysis forces doubting this point of view. The sharp, cleave-looking edges of the MCC particles suggesting the initial cellulose fibers were fractured; hence the hydrolysis made them brittle. Zhurkov showed that mechanical stress decreases the activation energy of the polymer fracture, which correlates with the elevated enthalpy of the MCC thermal destruction compared to the initial cellulose.

## 1. Introduction

Cellulose is widely used in the modern industry either in the native form or after deep chemical and/or mechanical treatment. Some branches use the purified and bleached cellulose, such as paper production [1], while others require the products of the deep chemical treatment of cellulose: nitrocellulose for propellants and coatings production [2,3], oxycellulose in pharmaceutics [4], viscose or MNMO solutions for fabrics production [5,6], etc. On the other hand, some of the products of the so-called mechano-chemical treatment of cellulose [7] have nearly the same chemical composition as a feedstock. Such materials are microcrystalline cellulose (MCC) and nanocellulose (NC), widely used in the modern industry. MCC is used as reinforcing material [8], in the food industry [9], and medicine [10]. NC is also used as reinforcing material, a base for films [11,12], and conductive materials’ production, as a rheological modifier (thickener), and for drug delivery [13,14].

MCC is pure, highly-crystalline cellulose, composed of shortened fragments of the original fibers [15]. The classical MCC production method consists of the treatment of the lignin-free and whitened cellulose by sulfuric or muriatic acid (2–3 h) at elevated temperature (100–150 °C) with a solvent-to-pulp ratio greater than 10 [8,9]. The nature of the acid does not affect the particle size [16,17], but affects the crystallinity index of the final product [17]. Simultaneously, the particles’ size decreased with the increase of the acid hydrolysis intensity [18]. The particles’ size does not depend significantly on the cellulose origin and the lignin removal technique [19]. However, the thermal stability and crystallinity of the MCC depend a bit on the delignification method [20,21]. In the course of acid hydrolysis, the degree of polymerization (DP) of the initial cellulose decreases by a factor of 5–10 [15,18,19]. The hydrolyzed pulp is neutralized, cleaned, and subjected to mechanical grinding. After drying, the pulp becomes a white homogeneous powder known as MCC [8,9].

The product of deeper cellulose grinding is known as nanocellulose (NC), which can be of two types: cellulose nanofibrils (CNFs) and cellulose nanocrystals (CNCs), each having different properties [13,22]. The CNF pulp is composed of fibrils with a cross-section of about 5–100 nm and a length of several microns, entangled with each other, that significantly increase the viscosity of CNF suspensions [22,23,24,25]. The portion of the crystalline fraction in CNF is relatively low and amounts to 40–60% depending on the method of manufacture [23,24]. Simultaneously, DP decreases by about one and a half to two times, compared with the values typical for the initial feedstock [24]. In contrast to CNF, the share of the crystalline fraction in CNC is high and often exceeds 90%, whereas particles have a smaller cross-section of about 5–20 nm and a length of 100–500 nm [22,23,25]. Such differences in the structure of the described nanomaterials correlate with the differences in the methods of their production [25]. More or less, all CNF production methods include the stage of the chemical pretreatment (TEMPO oxidation or enzymatic hydrolysis), followed by intense mechanical grinding: high-pressure homogenization, ultra-fine grinding, or microfluidization [24]. For the CNC production, lignin-free cellulose treated with relatively strong (40–80%) solutions of mineral acids at elevated temperature (40–80 °C) and then subjected to intensive mechanical grinding, such as ultrasonication or shear grinding [12,26]. The CNC production requires the rotor-stator gap to be about tens of microns, and the shaft rotation rate should be several thousand rpm, and dozens of processing cycles are required [12,24,25]. Power consumption for such a process could reach 30 kW/kg of the final product [25], which significantly increases its cost.

Manufacturing of both MCC and NC is rather laborious. It is accompanied by a noticeable (up to 70%) loss of mass, which decreases the yield of the final product and increases its cost [26]. Investigation of the wastewater generated in the MCC production course in our experiments revealed NC suspended in them, which can be extracted and used virtually without additional costs. The result can be directly used in the industry to obtain a useful supplemental product.

Acid hydrolysis is generally accepted as the main reason for the cellulose destruction in MCC production [8,9,19]. However, there exists another physically-based mechanism of polymer destruction proposed by Zhurkov [27,28,29]. The microcracks formed across mechanically stressed polymer fiber can efficiently promote its fracture. Moreover, the cracking intensifies in the stress concentration areas, which further concentrates the stress in these areas and provides for the avalanche-like increase in the number of cracks and the material fracture. The concentration of microcracks, the load applied, and the polymer’s breakdown directly correlate with each other, which was experimentally shown for several polymers, such as nylon, PE, PVC, and even for the cellulose derivatives [29]. Such a physics-of-failure-based model was successfully used for microelectronics reliability predictions [30] and analysis of the nitrocellulose thermal destruction process [31].

Zhurkov’s stress-based mechanism of polymer fracture can also affect the thermal properties of the polymer. Indeed, the internal stress’s energy would facilitate the thermal decomposition, while the stress-free fibers would be more stable. The TGA/DSC curves of raw cellulose and MCC have two peaks in the regions of 80–90 and 300–360 °C [8,32]. The first one is conditioned by the water evaporation, and its intensity directly correlates with the humidity of the sample. In contrast, the second peak appears due to the thermal decomposition of the material. On average, in raw cellulose, the second peak appears between 320 [15,17] and 350 °C [20,21], depending on the raw used. In MCC, the second peak can be found in a wide range of the temperatures (320–450 °C [33] either at greater temperatures [15,20,21,34] or lower temperatures [33] compared to the raw cellulose. However, the enthalpy of the thermal destruction of MCC was greater than raw cellulose [15].

**Figure A1:**
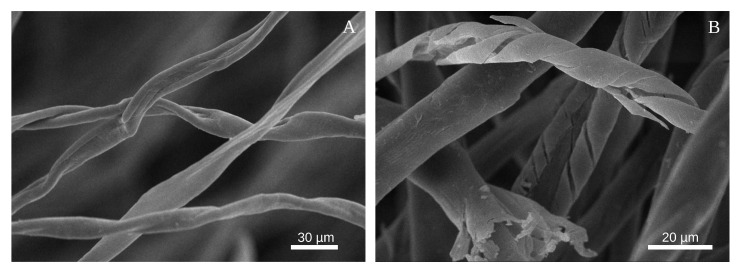
SEM images of the initial (**A**) and nitrated for 30 min (**B**) cotton cellulose. Cracking of the fibers reveals the helical structure invisible in the initial cellulose and in the MCC particles (Figure 2). For the details on the nitration process, please refer to [45].

Considering the processes taking place in the cellulose pulp is not conceivable without accounting for the details of its supramolecular structure. In the native cellulose, the polymer chains combine into the elementary fibrils (nanofibrils) having a diameter of 2.5 nm in wood and up to 20 nm in cellulose from tunicates [35,36,37,38]. Nanofibrils have a helical structure, but the helix pitch was not determined experimentally [39], while the molecular modeling brought the value of about 100 and 250 nm for the softwood and cotton fibrils, respectively [40]. In the cell wall, the nanofibrils are glued together by lignin and hemicelluloses [41] and form the helical microfibrils [42] having a thickness of about 30–40 nm and usually form bundles [43]. The microfibrils make up the cell wall [41,43], which also has a helical structure that becomes prominent in the cracked fibers (Figure A1). The helical structure is so typical for cellulose that it is realized even in the artificial fibers [44].

The helical pitch differs among the structural levels of cellulose organization due to the difference in their elements’ characteristic diameters. The twist direction sequentially changes when passing from the previous level to the next [45], caused by the energy gain provided by a decrease in the torsional stresses [46,47,48]. Due to the exponential dependence of the friction force on the number of turns in the coiled fiber, given by the Capstan equation, the supercoiled cellulose fibers cannot be disassembled without their untwisting [44,45,49]. This circumstance explains, for example, the preservation of the cellulose morphology even under the harsh conditions of the delignification and nitration processes [45].

## 2. Materials and Methods

### 2.1. Cellulose Processing

Cotton cellulose (UzCell, Uzbekistan) and bleached kraft hard-wood cellulose (Arkhangelsk paper and pulp mill, Russia) were used as raw materials.

**Figure 1:**
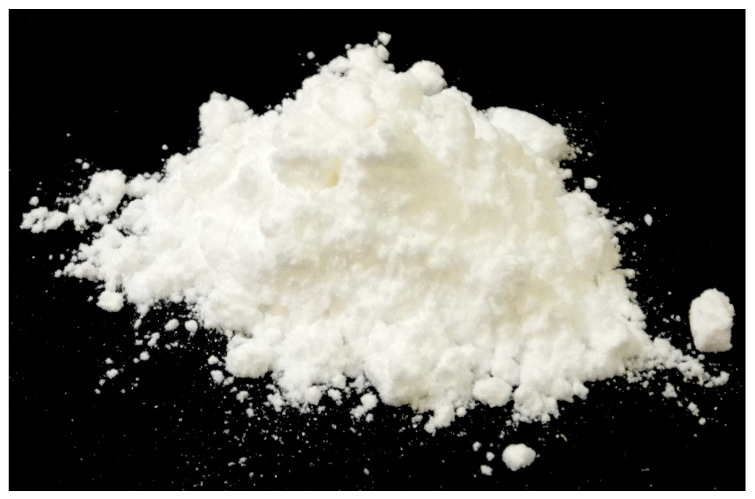
Microcrystalline cellulose (MCC) obtained from the hardwood cellulose raw according to the procedure described in the text.

At the first stage, the feedstock was soaked in distilled water at room temperature for 72 h (water consumption 30 kg/kg) and washed three times with fresh distilled water (water consumption 20 kg/kg). The resulting pulp was squeezed on the glass filter under vacuum (the resulting cellulose concentration was ∼12.5%). Then cellulose mass was treated with a hot (93–95 °C) 10% solution of sulfuric acid for 120 min (acid consumption 30 kg/kg). After the hydrolysis, the cellulose mass was squeezed again to remove the acid (the acid was recirculated), washed with distilled water until the neutral pH (water consumption 20 kg/kg), and dried with the warm air (90–95 °C) until the residual moisture content of 5–6%. The dried cellulose was shredded using a ball mill (BMR/60, LLC Tekhno-center, Rybinsk, Russia) and was not sonicated. The result was white, homogeneous, finely dispersed powder (Figure 1).

To harvest the NC suspension, the obtained MCC was soaked again into the distilled water and treated on a RIM-150 share grinder (gap ∼ 500 μm, shaft rotation speed 1250 rpm). The shear stress achieved by this machine was insufficient to convert MCC into NC [12,24], which allowed neglecting the generation of NC in the course of this treatment. The MCC particles were precipitated by centrifugation (10 min, 1300× *g*, Avanti J-15, Beckman Coulter, Brea, CA, USA), which resulted in the dense precipitate.

The NC films were obtained by drying the NC suspension on mica or glass surface at room temperature repeated up to 30 times to obtain thick films.

### 2.2. Analytical Approaches

The moisture content in the specimens was determined by the change in the sample’s mass after drying at 100 ± 5 °C, according to GOST 16932-93 (ISO/DIS 638-1/638-2). The specific surface area of MCC particles was determined according to the Brunauer–Emmett–Teller theory by the low-temperature (77 K) argon adsorption [50]. A comparative analysis of the MCC particle sizes was performed on a Fritsch Analysette 22 MicroTec plus instrument (0.08–2000 μm).

The degree of crystallinity was determined using the “peak height” method [51]. X-ray diffraction analysis of the obtained MCC samples was performed on a URD-6 diffractometer (Seifert-FPM, Freiberg, Germany) for medium and large angles at the accelerating voltage of 35 kV and current of 25 mA. The sample rotation speed was 1 min${}^{-1}$.

The IR spectra were recorded using a Tensor 27 spectrometer (Bruker, Billerica, MA, USA) with an incomplete internal reflection on a germanium crystal (resolution was 4 cm${}^{-1}$). The samples were taken every 20 min during the entire acid hydrolysis process. Before spectra registration, the samples were incubated for 72 h, at a relative humidity of 60 ± 5% and temperature of 22 ± 2 °C. The spectra were normalized to the absorption band 1056 cm${}^{-1}$.

The Solver HV microscope (LLC NT-MDT, Moscow, Russia) was used for the atomic force microscopy (semi-contact mode, room temperature, and atmospheric pressure). Solver HV was equipped with the standard cantilevers (LLC NT-MDT, Russia) with the natural frequencies of 110–180 kHz and a tip radius of 10 nm. TGA/DSC 3+ combined machine (Mettler Toledo, Greifensee, Switzerland) was used for thermogravimetric analysis and differential scanning calorimetry of cellulose. The samples (40 mg) were heated in the Al${}_{2}$O${}_{3}$ cup (300 μl) from 25 to 300 °C (5 °C/min) in the nitrogen atmosphere.

## 3. Results

### 3.1. Characterization of MCC

**Figure 2:**
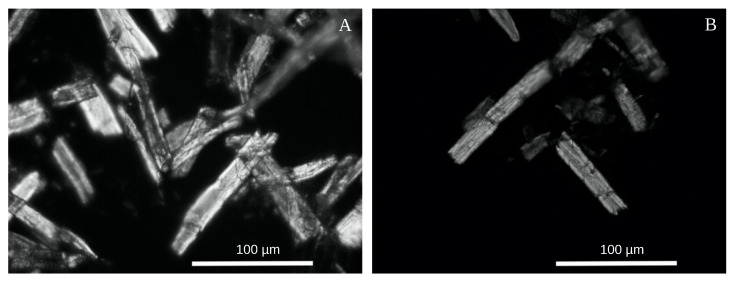
Dark-field micrographs of MCC particles obtained after single (**A**) and double (**B**) hydrolysis and subsequent grinding.

**Figure 3:**
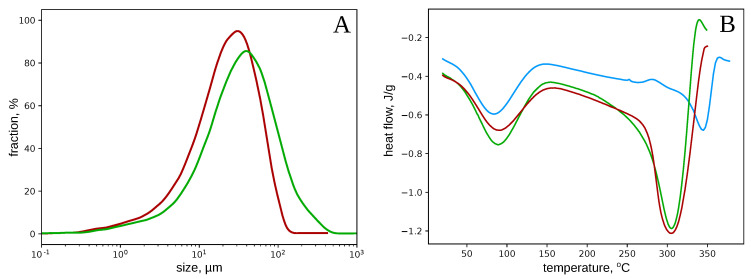
The size distribution of the MCC particles (**A**) and DSC-curves (**B**) for raw soft-wood cellulose (red line) and MCCs obtained after a single (green line) and double (red line) cycles of acid hydrolysis and subsequent dispersion.

The microcrystalline cellulose obtained in our technical process corresponded to the generally accepted standards [8,16]. The degree of crystallinity was 57 ± 1%, the specific surface area −4±1 m${}^{2}$/g, the bulk density −0.20±0.02 g/mL, and the moisture content −3.2±0.3%. The MCC particles were the cell fragments of 50–100 μm in length, with typical sharp edges (Figure 2) reported earlier [15,16,19]. The additional hydrolysis stage, followed by milling, did not significantly change the particle sizes. After the additional treatment, the maximum of MCC particles’ size distribution shifted from 40 to 30 μm (Figure 3A).

DSC experiments (Figure 3B) revealed a significant difference between the initial raw and MCC. In our case, the raw cellulose decomposition peak was found at 345, while that for MCCs was at 305 °C. According to the previous reports, the enthalpy of the raw cellulose thermal decomposition was 270±10 J/g, while that of MCCs was 690 and 780±10 J/g, which is two times greater than the reported earlier values [15]. So, the thermal stability of MCC was lower than initial raw, while the energy required for MCC decomposition was about three times greater. It should be noted that in the region 150–200 °C (between the peaks), the heat influx (and heat capacity) of MCCs (0.45±0.02 and 0.48±0.02) was also greater than of the initial cellulose (−0.36±0.01 mW/mg).

**Figure 4:**
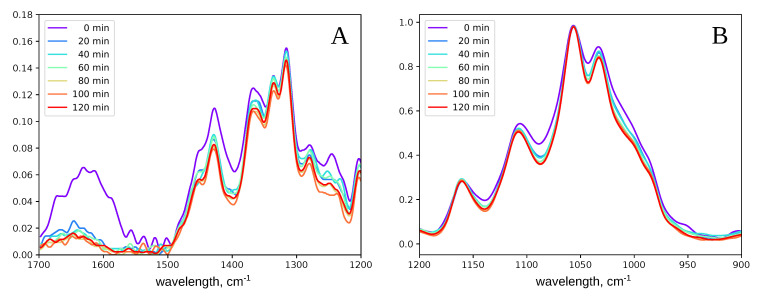
Changes in the IR spectra of cotton cellulose in the course of the acid hydrolysis. The spectrum was split into two parts: 1700–1200 cm${}^{-1}$ (**A**) and 1200–900 cm${}^{-1}$ (**B**).

In the IR spectra, the main changes occurred during the first 20 min of the acid hydrolysis. First of all, the intensity decreased in two bands in the range 1700–1500 cm${}^{-1}$ (Figure 4A) and 1020–950 cm${}^{-1}$ (Figure 4B). The absorption in the 1700–1500 cm${}^{-1}$ region belonged to the hemicelluloses’ carbonyl groups [52] and aromatic rings of lignin [53]. Therefore, both hemicelluloses and lignin were washed out in the first 20 min. However, it took at least 1 hour for the cellulose to acquire the homogeneity, typical for the MCC suspensions. Thus, the hydrolysis of cellulose itself required a longer time than hemicelluloses and lignin washing out.

### 3.2. Nanocellulose

**Figure 5:**
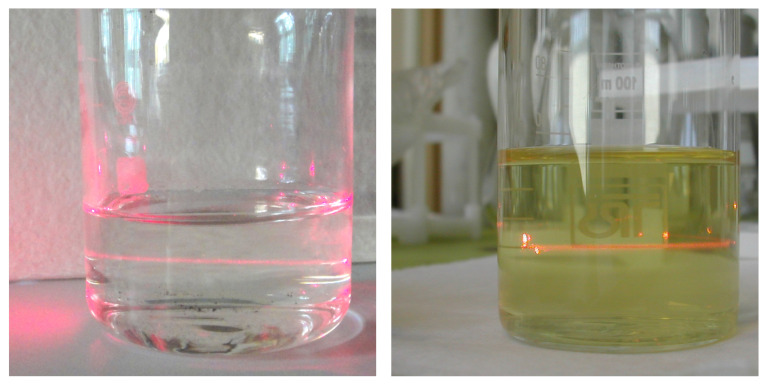
Light scattering (laser beam, 650 nm) by the sulfuric acid solution after a single cycle of the cellulose hydrolysis (**left**) and by the supernatant obtained after MCC mild mechanical treatment and centrifugation (**right**).

The intense light scattering was found in the sulfuric acid solution after a single cellulose hydrolysis cycle and the complete removal of the MCC particles by centrifugation at 1300× *g* (Figure 5, left). A similar fraction seemed to adsorb on the MCC particles, as the mild mechanical treatment of MCC by the share grinder also allowed obtaining the suspension of some fine fraction. The latter also remained in the supernatant after the centrifugation under the same conditions (Figure 5, right).

**Figure 6:**
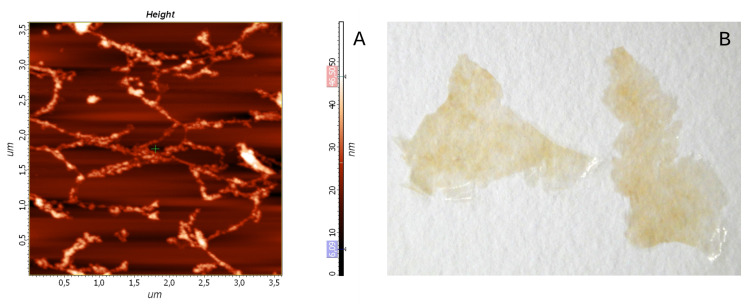
The atomic force microscopy (AFM) image of pseudofibrils, forming the “thin” film obtained by drying the MCC washout on the mica surface (**A**). The “thick” film obtained by 30-fold repeating the supernatant drying operation (**B**).

The weight content of nano-particles in the supernatant obtained after the MCC mild mechanical treatment was about $10^{-5}$. Drying the supernatant on the glass or mica surface resulted in a thin film, inseparable from the substrate and invisible to the eye. At such a low concentration, the nano-particles did not form a continuous layer, which allowed distinguishing between individual elements of the film by atomic force microscopy (Figure 6A). The thin films were composed of the helical cellulose pseudofibrils having about 100 nm in diameter and up to tens of microns in length. Manyfold repeating the watering-drying operation resulted in the continuous and uniform film visible to the eye (Figure 6B).

## 4. Discussion

MCC is usually considered an intermediate on the way to the NC production [32,54]. In this regard, much attention is paid to the process of further intensive mechanical MCC grinding. However, our findings indicate that the nanocellulose formes spontaneously in the process of MCC production. A relatively small amount of NC (about 0.03–0.05% of the initial weight) forms in the course of the hydrolysis of cellulose by a dilute sulfuric acid solution. According to the atomic force microscopy (AFM) data (Figure 6A), the obtained NC was represented by the cellulose nanofibrils (CNFs) since the observed elements had a considerable length, a diameter of about 100 μm, and were intertwined with each other [22,23,24,25].

**Figure A2:**
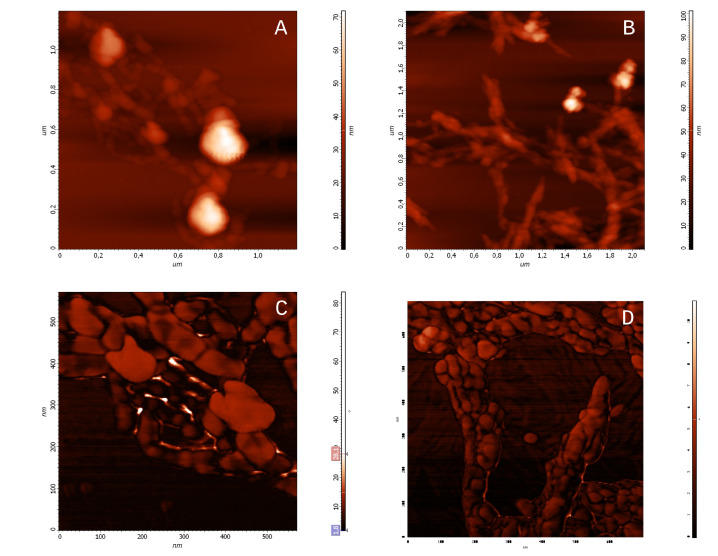
AFM images of the MCC obtained in a single (**A**,**C**) and double (**B**,**D**) cycles of acid hydrolysis and milling.

Herewith, the MCC production is considered a well-studied process of the acid hydrolysis of cellulose. However, if the MCC forms only due to simple hydrolysis, then the repeated treatment would have to reduce significantly the size of MCC particles, which was not observed (Figure 2, Figure 3A, and Figure A2). On the other hand, the nano- and microfibrils are twisted around each other and form a rather dense supercoiled structure that must be untwisted in the course of cellulose swelling in the acids [45,49]. The untwisting and subsequent twisting back and compaction of the cellulose fibrils allow explaining and managing the cellulose films’ properties [12], artificial cellulose fibers [44], and quality of nitrocellulose [45]. Following this hypothesis, we suppose the cellulose fibers are to be stressed in the twisting back after the acid removal. The accumulated mechanical stress can trigger the stress-based mechanism of the polymers fracture [27,30] under even a gentle mechanical treatment, as the additional load would summarize with the accumulated stress that would decrease the shear-stress threshold necessary for fibers fragmentation. Indeed, the procedure applied to the hydrolyzed pulp is far insufficient for noticeable breaking of the initial raw, while the acid-treated cellulose crumbles into dust.

According to Zhurkov’s equation [27,30], the activation energy for the destruction of polymer chains in the places of stress localization decreases by ΔE:

$$\Delta E=\nu \sigma =E_{a}-k_{B}T\;ln\frac{\tau }{\tau_{0}}$$

where τ is a fracture time, $\tau_{0}\approx 10^{-13}$ s, $E_{a}$ is an activation energy, σ is the characteristic value of structural stresses, ν is the stress sensitivity coefficient, $k_{B}$ is the Boltzmann constant, and *T* is a temperature. Thus, structural stresses can reduce the activation energy of destruction by 30–40 kJ/mol [55], which would increase the rate constant of destruction by two to three orders of magnitude.

In contrast, the stress-based hypothesis explains the stability of the MCC particles’ size in repeated hydrolysis-milling cycles. Indeed, the mechanical stress appeared in the twisting back process, and its distribution along the fiber correlates with the pitch of the helices presented in the fiber. Therefore, the distance between the neighboring fracture points would be greatest among sufficient for the relaxation of the accumulated mechanical stress below some critical threshold necessary to initiate Zhurkov’s fracture. Otherwise, the destruction would continue, and we would observe smaller pieces having a length short enough for stress relaxation. Accordingly, the repeated acid treatment of the short enough fragments does not lead to stress accumulation and further mechanical destruction of cellulose under the mild grinding.

Zhurkov’s fracture hypothesis also explains the straight, cleaved faces of the MCC particles (Figure 2) described by many other authors [15,16,19,20,33]. Under the load, the stress concentrates at the crack’s edges, which may result in its avalanche-like expansion and breaking of the fiber along it. In this case, the microcrack in the fiber’s weakest point would start to expand first, which would reduce the overall stress and preserve the other microcracks from expansion. Therefore, according to our hypothesis, the cellulose fibers break along such the weakest microcracks, and we observe their edges in the form of the MCC particle edges.

The thermal properties of the obtained MCC (Figure 3B) differed from the reported earlier. First of all, the MCC’s thermal decomposition temperature was rather low (∼305 °C) compared to the results of other groups [15,20,21,34]. The only reason for such an inconsistency could be the difference in the MCC production protocols. The critical step was probably using caustic soda for the acid neutralization after hydrolysis that we did not use. MCC is known to dissolve in alkalis, so even a short treatment with 1 N NaOH [15,17,20,21] could affect the cellulose structure and cause an increase in the decomposition temperature. Taking the rates of the cellulose and MCC thermal destruction to be approximately equal, the difference in the temperatures of decomposition allows rough accessing the difference in activation energies (Ea) of this process:

$$Ea_{MCC}=Ea_{cellulose}\frac{T_{MCC}}{T_{cellulose}}\approx 0.88\cdot Ea_{cellulose}$$

Variations of the destruction activation energy of 10–15% can be caused by the structural reorganization (or stress) of the polymer matrix that was shown for nitrocellulose [31]. Besides that, the enthalpy of the MCC decomposition process (−600–700 J/g) was two-to-three times greater than that of initial cellulose (∼−270 J/g), which qualitatively corresponds to the earlier results: the MCC thermal destruction always requires greater energy than initial raw [15,17,20,21,32,33,34]. Such a ratio confirms our hypothesis. Indeed, if the initial raw was a bit stressed, and in MCC, the mechanical stress relaxed, then the enthalpy of the raw cellulose decomposition would decrease exactly on the value of the stress’s energy.

## 5. Conclusions

Summarizing the observations made, we can propose that the MCC formation cannot be considered an exclusively chemical process. The insignificant decrease in the particles’ size after the repeated hydrolysis-and-milling cycle directly indicates this proposition. Indeed, the hydrolysis should intensify with the decrease of the cellulose fragments’ size, and after the repeater acid treatment, the particles’ size should decrease qualitatively. However, this is not the case. Based on the shape of the MCC particles and the native cellulose’s superhelical structure, we supposed that the fragmentation of the initial long cellulose fibers into micron-sized MCC particles occurs due to the mechanical stress accumulated upon the swelling and subsequent compactization of the superhelical cellulose during acid hydrolysis and washing-out. Mechanical stress can make the polymer structure brittle, which was described by Zhurkov [27]. The shape of the MCC particles and the gentleness of the necessary milling directly indicates the fragility of the cellulose after the acid hydrolysis and reinforce our hypothesis. Our proposition allows modification of the existing technology or even creating some novel methods of cellulose fragmentation. So, the main goal would be to increase the fibers’ stress, which would inevitably cause smaller fragments after the milling.

## References

[1] Gharehkhani S., Sadeghinezhad E., Kazi S.N., Yarmand H., Badarudin A., Safaei M.R., Zubir M.N.M. (2015). Basic Effects of Pulp Refining on Fiber Properties—A Review. Carbohydr. Polym..

[2] Braconnot H. (1833). De la Transformation de Plusieurs Substances Végétales en un Principe Nouveau. Ann. Chim. Phys..

[3] Saunders C., Taylor L. (1990). A Review of the Synthesis, Chemistry and Analysis of Nitrocellulose. J. Energ. Mat..

[4] Martina B., Kateřina K., Miloslava R., Jan G., Ruta M. (2009). Oxycellulose: Significant Characteristics in Relation to its Pharmaceutical and Medical Applications. Adv. Polym. Technol..

[5] Cross C., Bevan E. (1892). Improvements in Dissolving Cellulose and Allied Compounds. Br. Pat. Appl..

[6] Sayyed A.J., Deshmukh N.A., Pinjari D.V. (2019). A Critical Review of Manufacturing Processes Used in Regenerated Cellulosic Fibres: Viscose, Cellulose Acetate, Cuprammonium, LiCl/DMAc, Ionic Liquids, and NMMO Based Lyocell. Cellulose.

[7] Kuga S., Wu M. (2019). Mechanochemistry of Cellulose. Cellulose.

[8] Trache D., Khimeche K., Mezroua A., Benziane M. (2016). Physicochemical Properties of Microcrystalline Nitrocellulose from Alfa Grass Fibres and its Thermal Stability. J. Therm. Anal. Calorim..

[9] Nsor-Atindana J., Chen M., Goff H.D., Zhong F., Sharif H.R., Li Y. (2017). Functionality and Nutritional Aspects of Microcrystalline Cellulose in Food. Carbohydr. Polym..

[10] Kamel S., Ali N., Jahangir K., Shah S., El-Gendy A. (2008). Pharmaceutical Significance of Cellulose: A review. EXPRESS Polym. Lett..

[11] Nogi M., Iwamoto S., Nakagaito A.N., Yano H. (2009). Optically Transparent Nanofiber Paper. Adv. Mater..

[12] Zlenko D.V., Nikolsky S.N., Vedenkin A.S., Politenkova G.G., Skoblin A.A., Melnikov V.P., Mikhaleva M.G., Stovbun S.V. (2019). Twisting of Fibers Balancing the Gel–Sol Transition in Cellulose Aqueous Suspensions. Polymers.

[13] Du X., Zhang Z., Liu W., Deng Y. (2017). Nanocellulose-Based Conductive Materials and Their Emerging Applications in Energy Devices—A Review. Nano Energy.

[14] Kargarzadeh H., Mariano M., Huang J., Lin N., Ahmad I., Dufresne A., Thomas S. (2017). Recent Developments on Nanocellulose Reinforced Polymer Nanocomposites: A Review. Polymer.

[15] Trache D., Donnot A., Khimeche K., Benelmir R., Brosse N. (2014). Physico-Chemical Properties and Thermal Stability of Microcrystalline Cellulose Isolated from Alfa Fibres. Carbohydr. Polym..

[16] El-Sakhawy M., Hassan M.L. (2007). Physical and Mechanical Properties of Microcrystalline Cellulose Prepared from Agricultural Residues. Carbohydr. Polym..

[17] Tarchoun A.F., Trache D., Klapötke T.M., Derradji M., Bessa W. (2019). Ecofriendly Isolation and Characterization of Microcrystalline Cellulose from Giant Reed Using Various Acidic Media. Cellulose.

[18] Vanhatalo K.M., Dahl O.P. (2014). Effect of Mild Acid Hydrolysis Parameters on Properties of Microcrystalline Cellulose. BioResources.

[19] Leppänen K., Andersson S., Torkkeli M., Knaapila M., Kotelnikova N., Serimaa R. (2009). Structure of Cellulose and Microcrystalline Cellulose from Various Wood Species, Cotton and Flax Studied by X-ray Scattering. Cellulose.

[20] Beroual M., Boumaza L., Mehelli O., Trache D., Tarchoun A.F., Khimeche K. (2020). Physicochemical Properties and Thermal Stability of Microcrystalline Cellulose Isolated from Esparto Grass Using Different Delignification Approaches. J. Polym. Environ..

[21] Beroual M., Trache D., Mehelli O., Boumaza L., Tarchoun A.F., Derradji M., Khimeche K. (2020). Effect of the Delignification Process on the Physicochemical Properties and Thermal Stability of Microcrystalline Cellulose Extracted from Date Palm Fronds. Waste Biomass Valoriz..

[22] Abitbol T., Rivkin A., Cao Y., Nevo Y., Abraham E., Ben-Shalom T., Lapidot S., Shoseyov O. (2016). Nanocellulose, a Tiny Fiber with Huge Applications. Curr. Opin. Biotechnol..

[23] Moon R.J., Martini A., Nairn J., Simonsen J., Youngblood J. (2011). Cellulose Nanomaterials Review: Structure, Properties and Nanocomposites. Chem. Soc. Rev..

[24] Qing Y., Sabo R., Zhu J., Agarwal U., Cai Z., Wu Y. (2013). A Comparative Study of Cellulose Nanofibrils Disintegrated via Multiple Processing Approaches. Carbohydr. Polym..

[25] Khalil H.A., Davoudpour Y., Islam M.N., Mustapha A., Sudesh K., Dungani R., Jawaid M. (2014). Production and Modification of Nanofibrillated Cellulose Using Various Mechanical Processes: A review. Carbohydr. Polym..

[26] Bondeson D., Mathew A., Oksman K. (2006). Optimization of the Isolation of Nanocrystals from Microcrystalline Cellulose by Acid Hydrolysis. Cellulose.

[27] Zhurkov S., Korsukov V. (1974). Atomic Mechanism of Fracture of Solid Polymers. J. Polym. Sci. Polym. Phys. Ed..

[28] Zhurkov S. (1984). Kinetic Concept of the Strength of Solids. Int. J. Fract..

[29] Zhurkov S., Kuksenko V. (1975). The Micromechanics of Polymer Fracture. Int. J. Fract..

[30] Suhir E. (2014). Three-Step Concept (TSC) in Modeling Microelectronics Reliability (MR): Boltzmann-Arrhenius-Zhurkov (BAZ) Probabilistic Physics-of-Failure Equation Sandwiched between Two Statistical Models. Microelectron. Reliab..

[31] Stovbun S.V., Lomakin S., Shchegolikhin A., Skoblin A., Melnikov V. (2018). Role of Structural Stresses in the Thermodestruction of Supercoiled Cellulose Macromolecules after Nitration. Russ. J. Phys. Chem. B.

[32] Satyamurthy P., Vigneshwaran N. (2013). A Novel Process for Synthesis of Spherical Nanocellulose by Controlled Hydrolysis of Microcrystalline Cellulose Using Anaerobic Microbial Consortium. Enzym. Microb. Technol..

[33] Rasheed M., Jawaid M., Karim Z., Abdullah L.C. (2020). Morphological, Physiochemical and Thermal Properties of Microcrystalline Cellulose (MCC) Extracted from Bamboo Fiber. Molecules.

[34] Kuthi F.A.A., Norzali N.R.A., Badri K.H. (2016). Thermal Characteristics of Microcrystalline Cellulose from Oil Palm Biomass. Malays. J. Anal. Sci..

[35] Hult E., Larsson P., Iversen T. (2001). Cellulose Fibril Aggregation – an Inherent Property of Kraft Pulps. Polymer.

[36] Jarvis M. (2003). Cellulose Stacks Up. Nature.

[37] Newman R. (1999). Estimation of the Lateral Dimensions of Cellulose Crystallites Using ^13^C NMR Signal Strengths. Sol. St. Nucl. Magn. Reson..

[38] Zhao Y., Li J. (2014). Excellent Chemical and Material Cellulose from Tunicates: Diversity in Cellulose Production Yield and Chemical and Morphological Structures from Different Tunicate Species. Cellulose.

[39] Fernandes A., Thomas L., Altaner C., Callow P., Forsyth V., Apperley D., Kennedy C., Jarvis M. (2011). Nanostructure of Cellulose Microfibrils in Spruce Wood. Proc. Natl. Acad. Sci. USA.

[40] Zhao Z., Shklyaev O., Nili A., Mohamed M., Kubicki J., Crespi V., Zhong L. (2013). Cellulose Microfibril Twist, Mechanics, and Implication for Cellulose Biosynthesis. J. Phys. Chem. A.

[41] O’Sullivan A.C. (1997). Cellulose: The Structure Slowly Unravels. Cellulose.

[42] Hanley S.J., Revol J.F., Godbout L., Gray D.G. (1997). Atomic Force Microscopy and Transmission Electron Microscopy of Cellulose from *Micrasterias Denticulata*; Evid. A Chiral Helical Microfibril Twist. Cellulose.

[43] Hanley S.J., Giasson J., Revol J.F., Gray D.G. (1992). Atomic Force Microscopy of Cellulose Microfibrils: Comparison with Transmission Electron Microscopy. Polymer.

[44] Usachev S.V., Zlenko D.V., Nagornova I.V., Koverzanova E.V., Mikhaleva M.G., Vedenkin A.S., Vtyurina D.N., Skoblin A.A., Nikolsky S.N., Politenkova G.G. (2020). Structure and Properties of Helical Fibers Spun from Cellulose Solutions in [Bmim]Cl. Carbohydr. Polym..

[45] Nikolsky S.N., Zlenko D.V., Melnikov V.P., Stovbun S.V. (2019). The Fibrils Untwisting Limits the Rate of Cellulose Nitration Process. Carbohydr. Polym..

[46] Tverdislov V., Malyshko E., Il’chenko S., Zhulyabina O., Yakovenko L. (2017). A Periodic System of Chiral Structures in Molecular Biology. Biophysics.

[47] Malyshko E., Murtazina A., Tverdislov V. (2020). Chirality as a Physical Basis of Hierarchical Periodization of Biomacromolecular Structures. Biophysics.

[48] Tverdislov V.A., Malyshko E.V. (2020). Chiral Dualism as an Instrument of Hierarchical Structure Formation in Molecular Biology. Symmetry.

[49] Stovbun S.V., Nikolsky S.N., Melnikov V.P., Mikhaleva M.G., Litvin Y., Shchegolikhin A., Zlenko D.V., Tverdislov V., Gerasimov D., Rogozin A. (2016). Chemical Physics of Cellulose Nitration. Russ. J. Phys. Chem. B.

[50] Zografi G., Kontny M., Yang A., Brenner G. (1984). Surface Area and Water Vapor Sorption of Microcrystalline Cellulose. Int. J. Pharm..

[51] Terinte N., Ibbett R., Schuster K.C. (2011). Overview on Native Cellulose and Microcrystalline Cellulose I Structure Studied by X-ray Diffraction (WAXD): Comparison between Measurement Techniques. Lenzing. Berichte.

[52] Delbecq F., Wang Y., Muralidhara A., Ouardi K.E., Marlair G., Len C. (2018). Hydrolysis of Hemicellulose and Derivatives—A Review of Recent Advances in the Production of Furfural. Front. Chem..

[53] Duval A., Lawoko M. (2014). A Review on Lignin-Based Polymeric, Micro- and Nano-Structured Materials. React. Funct. Polym..

[54] Shankar S., Rhim J.W. (2016). Preparation of Nanocellulose from Micro-Crystalline Cellulose: The Effect on the Performance and Properties of Agar-Based Composite Films. Carbohydr. Polym..

[55] Stovbun S.V., Skoblin A.A., Zlenko D.V. (2018). Self Assembly and Gelation in Solutions of Chiral N-trifluoroacetylated *α*-aminoalcohols. Chem. Phys..

